# Comparing the efficacy of traditional Chinese exercises and general aerobic exercises in university students with sleep disorders: A systematic review and meta-analysis

**DOI:** 10.1097/MD.0000000000038521

**Published:** 2024-06-07

**Authors:** Zhihui Yang, Haiting Zhai, Zhiwei Yang, Boxuan Ning

**Affiliations:** a Beijing Sport University, Beijing, China; b Sports Coaching College, Beijing Sport University, Beijing, China; c School of Basic Sciences for Aviation, Naval Aviation University, Yantai, China.

**Keywords:** aerobic exercise, sleep disorders, Traditional Chinese exercise, university students

## Abstract

**Background:**

The objective of this study was to compare the impact of traditional Chinese exercise (TCEs) and general aerobic exercise (GAEs) on the sleep quality of university students and to determine which exercise is more effective in improving sleep quality in this specific population.

**Methods:**

We utilized Review Manager 5.3 to analyze data from 21 randomized controlled trials (RCTs), which included a total of 1252 participants. Effect sizes of TCEs and GAEs were compared using a random-effects model. Subgroup analyses were conducted on 3 modulating variables: times per session, frequency per week, and period.

**Results:**

A Meta-analysis of 14 RCTs showed that both TCEs (standard mean difference [SMD] = −0.89, 95% CI: −1.18 to −0.61; *P* < .00001) and GAEs (SMD = −1.53, 95% CI: −2.10 to −0.97; *P* < .00001) can significantly improve the sleep of university students, with a significant difference between TCEs and GAEs (*P* = .05). Both GAEs and TCEs had positive effects on various aspects of sleep quality, including subjective sleep quality, sleep latency, sleep duration, habitual sleep efficiency, sleep disturbance, use of sleep medication, and daytime dysfunction. A subgroup analysis of aerobic exercise showed that the effect size was larger in the 40 to 60 minutes group compared to the 60 to 90 minutes group (SMD = −1.89; 95% CI: −2.19 to −1.59; *P* < .00001). Furthermore, the effect size was larger in the 3 to 5 times per week group compared to the 2 times per week group (SMD = −1.56; 95% CI: −2.33 to −0.80; *P* < .0001). The effect size was also found to be larger in a period of 2 to 4 weeks compared to 6 to 18 weeks (SMD = −1.85; 95% CI: −2.17 to −1.54; *P* < .00001).

**Conclusion:**

GAEs is more effective than TCEs in improving the sleep quality of university students. An optimal aerobic exercise regimen for enhancing sleep quality among university students involves engaging in sessions lasting 40~60 minutes, 3~5 times per week, over a duration of 4 weeks.

## 1. Introduction

Sleep quality is a comprehensive indicator that encompasses an individual sleep state, including factors such as the speed of falling asleep and the duration of deep sleep.^[1]^ It serves as an important measure of quality of life and is closely associated with stress, mood, and psychological health status.^[2–5]^ The college period, which plays a significant role in the psychosocial development of students, presents numerous challenges such as academic progress, employment, and establishing interpersonal relationships. Consequently, sleep quality issues often arise. Research indicates that approximately 25% to 35% of university students experience varying degrees of sleep disorders.^[6]^ These factors not only impact individuals’ physical health but also have a detrimental effect on their mental well-being. This can potentially lead to the development of depression and anxiety, which in turn can further hinder their academic achievement. Currently, the majority of sleep disorder patients improve their sleep quality through medication.^[7]^ While medication can effectively treat sleep disorders quickly, it can also lead to adverse reactions and dependence, and withdrawal can result in rebound effects. For university students who experience sleep problems but do not yet meet the clinical diagnostic criteria for sleep disorders, medication may not be the ideal intervention. Considering the growing interest in non-pharmacological interventions for sleep disorders and strategies to enhance physical health, many doctors and scholars have been actively researching these areas. Research has shown that traditional Chinese practices (TCEs) like Tai Chi and Qigong can have a positive impact on sleep quality by reducing stress and anxiety and promoting relaxation of both the mind and body.^[8,9]^ Tai Chi practice, with its slow, flowing movements and deep breathing techniques, may alleviate symptoms of insomnia and sleep disorders, as well as improve the ability to fall asleep and stay asleep.^[10]^ Studies suggest that practicing Qigong can lower anxiety levels and enhance mental health, ultimately leading to better sleep quality.^[11,12]^ TCEs have gained widespread attention for their unique movement patterns and breathing techniques, believed to help balance the mind and body, regulate qi and blood, and promote energy flow. General aerobic exercises (GAEs) is also important in the treatment of sleep disorders.^[13]^ Taheri et al found that morning exercise improves cognitive performance decrements caused by partial sleep deprivation in elite athletes.^[14]^ Morteza T and Fariba V conducted a study that found that GAEs has a positive impact on attention and sleep quality among professional volleyball players.^[15]^ GAEs has been shown to enhance sleep quality by reducing the time it takes to fall asleep, decreasing the frequency of nighttime awakenings, and promoting metabolic processes, cardiovascular function, and body temperature regulation. These factors collectively contribute to a more regulated sleep cycle and improved overall sleep quality. These studies suggest that both GAEs and TCEs are effective non-pharmacological therapies for improving sleep quality, offering simple and cost-effective solutions.^[16–19]^ However, current research primarily focuses on the middle-aged and elderly population in the context of TCEs.^[20]^ Additionally, the effectiveness of various intensities of GAEs in improving sleep disorders can vary.^[17,18]^ Moreover, there may be differences in the mechanisms through which TCEs and GAEs improve sleep disorders. There is a lack of research comparing the effectiveness of these 2 approaches in improving sleep disorders among university students. Therefore, this meta-analysis to compare the effectiveness of TCEs and GAEs in addressing sleep disorders among university students.

## 2. Research methodology

### 2.1. Literature search

**Figure 1.**
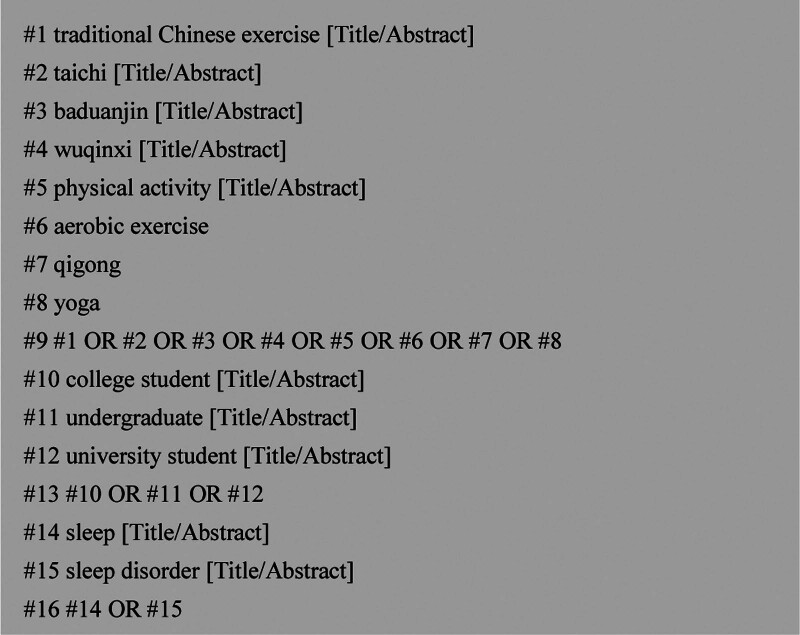
Using the PubMed database as an example, the process of searching through keywords was demonstrated.

Two searchers (YZH, ZHT) conducted the literature search in an independent double-blind manner, the Chinese literature was searched in the China Knowledge Network database, and the English literature was searched in the Web of Science, PubMed, Cochrane Library, the databases of Embase, Scopus from January 1, 2000 to December 4, 2023. The search was performed using combinations of subject headings and free words such as TCEs, taichi, baduanjin, wuqinxi, qigong, yoga, physical activity, aerobic training, college student, university student, undergraduate, sleep, sleep disorders. Taking the PubMed database search as an example. (1 traditional Chinese exercise [Title/Abstract] or 2 taichi [Title/Abstract] or 3 baduanjin [Title/Abstract] or # 4wuqinxi [Title/Abstract] or # 5 physical activity [Title/Abstract] or # 6 training [Title/Abstract] or # 7 qigong [Title/Abstract] or # 8 yoga [Title/Abstract]) and (# 9 student [Title/Abstract] or undergraduate physical activity [Title/Abstract]) and (sleep [Title/Abstract] or 12 sleep disorder [Title/Abstract]) was searched (Fig. 1). The retrieved literature from each database was imported into EndNote X 9 (Clarivate Analytics) for further primary screening and inclusion according to the Preferred Reporting Items for Systematic Reviews and Meta-Analyses 2020 guidelines for systematic reviews and meta-analysis.

### 2.2. Inclusion and exclusion criteria

#### 2.2.1. Inclusion criteria

Literature type: The included literatures were randomized controlled trials (RCTs); The subjects were all university students; The experimental group and the control group provided information on the sample size, mean value, and standard deviation of the subjects; The experimental intervention program was TCEs (taichi, baduanjin, wuqinxi, qigong, etc) or general GAEs (jog, yoga, baseball, roller skating, biking, baseball, walking, etc); The study encompassed the description and procedure of the particular experiment; The Pittsburgh Sleep Quality Index (PSQI) total score was chosen as the measure of outcome.

#### 2.2.2. Exclusion criteria

**Figure 2.**
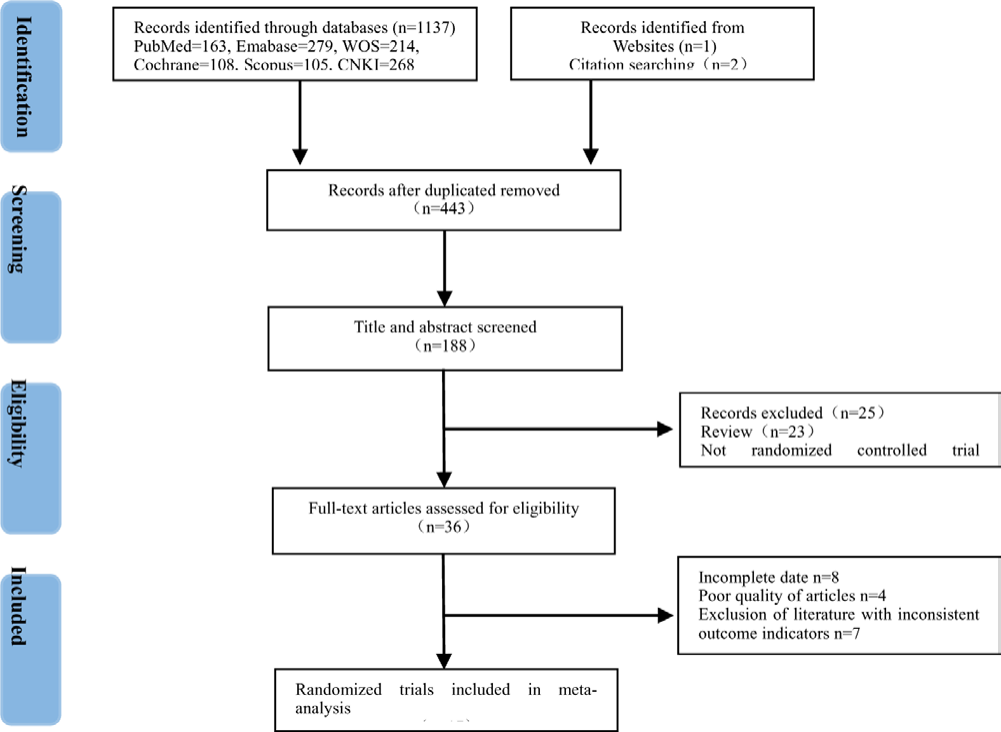
The flow of study selection.

Repeated literatures; Subjects are not university students; Subjects are university students majoring in physical education; Subjects are review literatures and systematic review literatures; Literatures without full text or unable to be obtained; Literatures with less rigorous experimental design and inconsistent outcome indicators; Non-Chinese and English literatures; Literatures that do not meet the inclusion criteria (Fig. 2).

### 2.3. Literature data extraction

In this study, 2 search personnel (YZH, ZHT) extracted and entered relevant indicators from the included literatures in an independent double-blind manner. Entered content: First author of the literature, publication time, gender, age, sample size of experimental and control groups, intervention content, exercise program (amount, intensity, frequency and cycle of exercise), outcome measures, etc. Disputes were discussed by a third author or group.

### 2.4. Literature quality assessment

**Figure 3.**
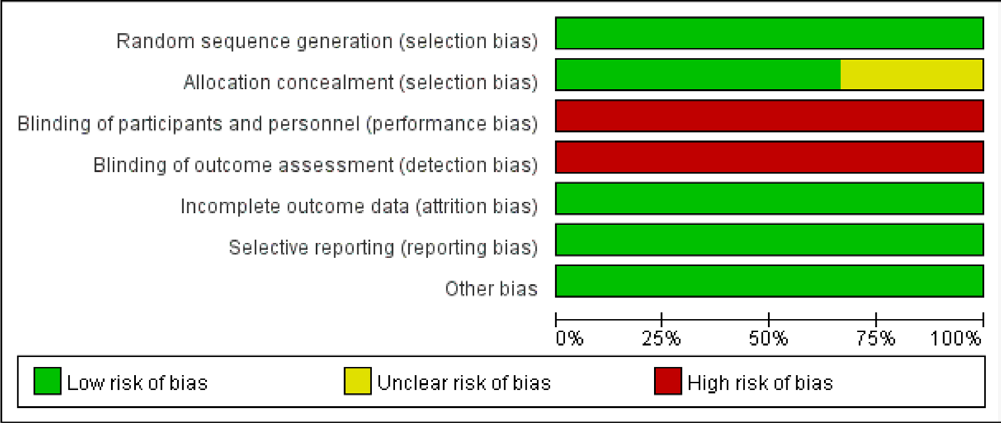
Diagram of the included literature along with its quality assessment is used to evaluate the included studies’ publication and their methodological quality.

**Figure 4.**
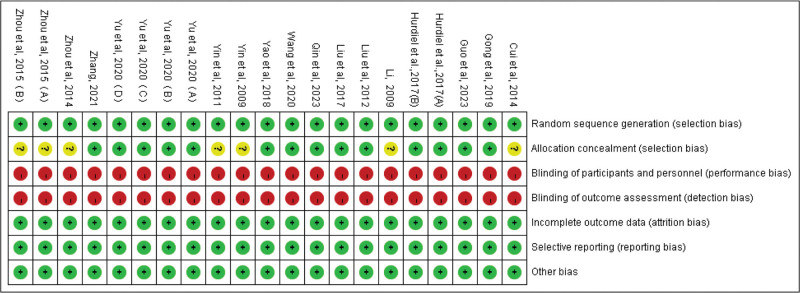
Summary diagram of the included literature with quality assessment is used to evaluate the included studies’ publication and their methodological quality.

In this study, the quality of the included articles was assessed using a modified version of the Cochrane Risk Bias Assessment Form (Figs. 3 and 4). The contents include: Cochrane Handbook consensus sequence generation (A), blinding (B), incomplete outcome data (C), selective outcome reporting (D) and other biases (E). Each article was evaluated as “yes,” “no,” and “unclear.” In this study, a scoring system was used, with 1 point for low risk, 0 point for A and B assessments if labeled “unclear” or “no,” and 0 point for C, D, and E assessments if labeled “yes” or “unclear.” The quality of literature was assessed based on a scoring system. A score of <3 was considered as low-quality literature, while a score of 3 to 4 was classified as medium quality literature. A score of 5 was considered as high-quality literature (Table 1).

**Table 1:** Literature quality evaluation form of Cochrane.

	A	B	C	D	E	Total score
Yu et al, 2021^[21]^	1	0	1	1	1	4
Zhou et al, 2015^[22]^	1	0	1	1	1	4
Zhou, 2014^[23]^	1	0	1	1	1	4
Yao et al, 2018^[24]^	1	0	1	1	1	4
Liu et al, 2012^[25]^	1	0	1	1	1	4
Qin et al, 2023^[26]^	1	0	1	1	1	4
Cui et al, 2014^[27]^	1	0	1	1	1	4
Wang et al, 2020^[28]^	1	0	1	1	1	4
Zhang, 2021^[29]^	1	0	1	1	1	4
Guo et al, 2023^[30]^	1	0	1	1	1	4
Hurdiel et al, 2017^[31]^	1	0	1	1	1	4
Li, 2009^[32]^	1	0	1	1	1	4
Liu., 2017^[33]^	1	0	1	1	1	4
Yin et al, 2009^[34]^	1	0	1	1	1	4
Yin et al, 2011^[35]^	1	0	1	1	1	4
Gong et al, 2019^[36]^	1	0	1	1	1	4

### 2.5. Data analysis and processing

The outcome measures of the included articles in this study were continuous variables, and the effect size measure used was the standard mean difference (SMD). An SMD value <0.2 is considered a small effect size, while a value between 0.2 and 0.5 is also considered a small effect size. A medium effect size is classified as a value between 0.5 and 0.8, and a large effect size is considered when the SMD value is 0.8 or greater.^[37]^ The magnitude of heterogeneity was quantitatively assessed with I^2^ values, and I^2^ < 40% was low heterogeneity, 40 ≤ I^2^ ≤ 70% was moderate heterogeneity, and I^2^ > 70% was high heterogeneity. When there was no heterogeneity or low heterogeneity, fixed effect model was used for meta-analysis, and random effect model was used for meta-analysis when heterogeneity was more obvious. Sensitivity analysis was performed to find the source of heterogeneity, and traditional funnel plots were performed to test publication bias.

## 3. Results

### 3.1. Basic information on literature inclusion

A total of 21 data from 16 RCTs with a total of 1252 subjects were included in this study.^[21–36]^ Eight studies were conducted in women^[21, 23, 31, 36]^ and another 13^[22, 24, 26–30, 33, 36]^ investigated a mix of men and women. The sample size included in the study ranged from 19 to 183 individuals, aged 17 to 25 years. The intervention in the exercise program contained: TCEs, including: Tai Chi, Qigong, Baduanjin, Wuqinxi; aerobic exercises, including: yoga, mass calisthenics, roller skating, cycling, jogging and other forms of exercise. The duration of each exercise was between 30 and 90 minutes, and the frequency of exercise was between 3 and 7 times a week. Period ranged from 2 to 18 weeks. Outcome measures were the Pittsburgh Sleep Quality Inventory (PSQI) scale (Table 2).

**Table 2:** Basic characteristics of included studies.

Author, yr	Sex	Age	Sample size	Intervention		Intervention (frequency and period)	Outcome
				Experimental group	Control group		
Yu et al, 2020 (A)	Female	18. 7	e.g.:23 CG:19	Taichi, Baduanjin, Wuqinxi	Non-intervention	30 min/session, 5 times/wk, 8 wk	PSQI
Yu et al, 2020 (B)	Female	18. 8	e.g.:18 CG:19	Taichi, Baduanjin, Wuqinxi	Non-intervention	60 min/session, 5 times/wk, 8 wk	PSQI
Yu et al, 2020 (C)	Female	18. 9	e.g.:23 CG:19	Taichi, Baduanjin, Wuqinxi	Non-intervention	30 min/session, 5 times/wk, 16 wk	PSQI
Yu et al, 2020 (D)	Female	18. 1	e.g.:18 CG:19	Taichi, Baduanjin, Wuqinxi	Non-intervention	60 min/session, 5 times/wk, 16 wk	PSQI
Zhou et al, 2015 (A)^[22]^	Female and male	18~24	e.g.:25 CG:25	Qigong	No intervention	60 min/session, 5 times/wk, 18 wk	PSQI
Zhou et al, 2015 (B)^[22]^	Female and male	18~25	e.g.:25 CG:25	Qigong	No intervention	90 min/session, 3 times/wk, 18 wk	PSQI
Zhou, 2014^[23]^	Female	NA	60	Taichi	Physical exercise	60 min/session, 3 times/wk, 16 wk	PSQI
Yao et al, 2018^[24]^	Female and male	18~22	110	Qigong	Cognitive education of sleep behavior	40 min/session, 3 times/wk, 18 wk	PSQI
Liu et al, 2012^[25]^	Female and male	NA	e.g.:30 CG:30	Baduanjin	Non-intervention	45–60 min/session, 5 times/wk, 10 wk	PSQI
Qin et al, 2023^[26]^	Female and male	e.g.:17. 50 ± 0. 71 CG:17. 60 ± 0. 84	e.g.:40 CG:40	Baduanjin	Routine activities	60 min/session, 3 times/wk, 12 wk	PSQI
Cui et al, 2014^[27]^	Female and male	18 ~ 24	e.g.:40 CG:40	Qigong·Liuzijue	Non special physical exercise	60 min/session, 5 times/wk, 8 wk	PSQI
Wang et al, 2020^[28]^	Female and male	NA	e.g.:30 CG:30	Wuqinxi	Non-intervention	40 min/session, 5 times/wk, 24 wk	PSQI
Zhang, 2021^[29]^	Female and male	NA	e.g.:30 CG:30	Traditional Physical Fitness	Non intervention	60 min/session, 5 times/wk, 12 wk	PSQI
Guo et al, 2023^[30]^	Female and male	e.g.:19. 60 ± 0. 88 CG:19. 60 ± 0. 88	e.g.:20 CG:20	Yoga	Routine activities	45 min/session，2 times/wk，4 wk	PSQI
Hurdiel et al, 2017 (A)^[31]^	Female	20. 1 ± 1.7	e.g.:10 CG:9	Roller skating, biking, baseball, walking/running	Non-intervention	90 min/session, 2 times/wk, 6 wk	PSQI
Hurdiel et al, 2017 (B)^[31]^	Female	20. 1 ± 1.8	e.g.:10 CG:10	Roller skating, biking, baseball, walking/running	Non intervention	90 min/session, 2 times/wk, 12 wk	PSQI
Li, 2009^[32]^	Female and male	17~24	e.g.:103 CG:80	Circuit	Keep a routine	90 min/session, times/wk, 18 wk	PSQI
Liu., 2017^[33]^	Female and male	20. 16 ± 1.37	e.g.:30 CG:30	Jogging	Non-intervention	50 min/session, 5 times/wk, 2 wk	PSQI
Yin et al, 2009^[34]^	Female and male	17~21	72	Masses aerobics and yoga	Non-intervention	60 min/session, 3 times/wk, 3 wk	PSQI
Yin et al, 2011^[35]^	Female and male	18~22	120	Masses aerobics	Non-intervention	45~60 min/session, 3 times/ week, 4 wk	PSQI
Gong et al, 2019^[36]^	Female	22.85 ± 1.26	e.g.:34 CG:36	Aerobic exercise and yoga	Non-intervention	40 min/session, 3 times/wk, 8 wk	PSQI

CG = control group, EG = experimental group, PSQI = Pittsburgh Sleep Quality Inventory.

### 3.2. Publication bias testing

**Figure 5.**
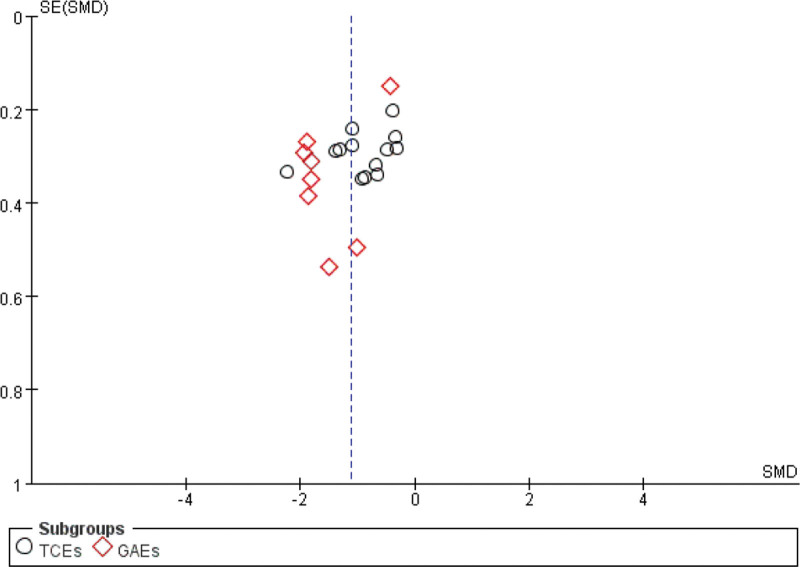
Funnel plots were used to assess publication bias or heterogeneity in studies.

The funnel plot analysis of the impact of physical exercise on sleep quality among university students revealed a predominantly symmetrical distribution of scatter points along the middle and upper axes. Only 2 scatter points were observed in the middle position, which exhibited slight deviation from other studies. The absence of significant publication bias among the studies and the reliability of the study findings were confirmed by the final results, indicating that this bias had no significant impact (Fig. 5).

### 3.3. Sensitivity analysis

Sensitivity analysis is a valuable technique used to assess the stability and reliability of the final results in systematic reviews or meta-analysis.^[37]^ Sensitivity analysis was performed on the data of 21 included studies by removing the literature one by one and transforming the effect model to observe the changes in the effect size recorded. It was found that Li, 2009^[32]^ in GAEs and Zhang, 2021^[29]^ in TCEs had a greater impact on heterogeneity, and after removing these 2 articles, heterogeneity I^2^ = 48 in TCEs, heterogeneity I^2^ = 0 in GAEs, the results of this study demonstrated no significant change in effect size, suggesting that the findings are highly credible. Therefore, both articles can be retained without any concerns.

### 3.4. Meta-analysis results

#### 3.4.1. Compared to total PSQI score

**Figure 6.**
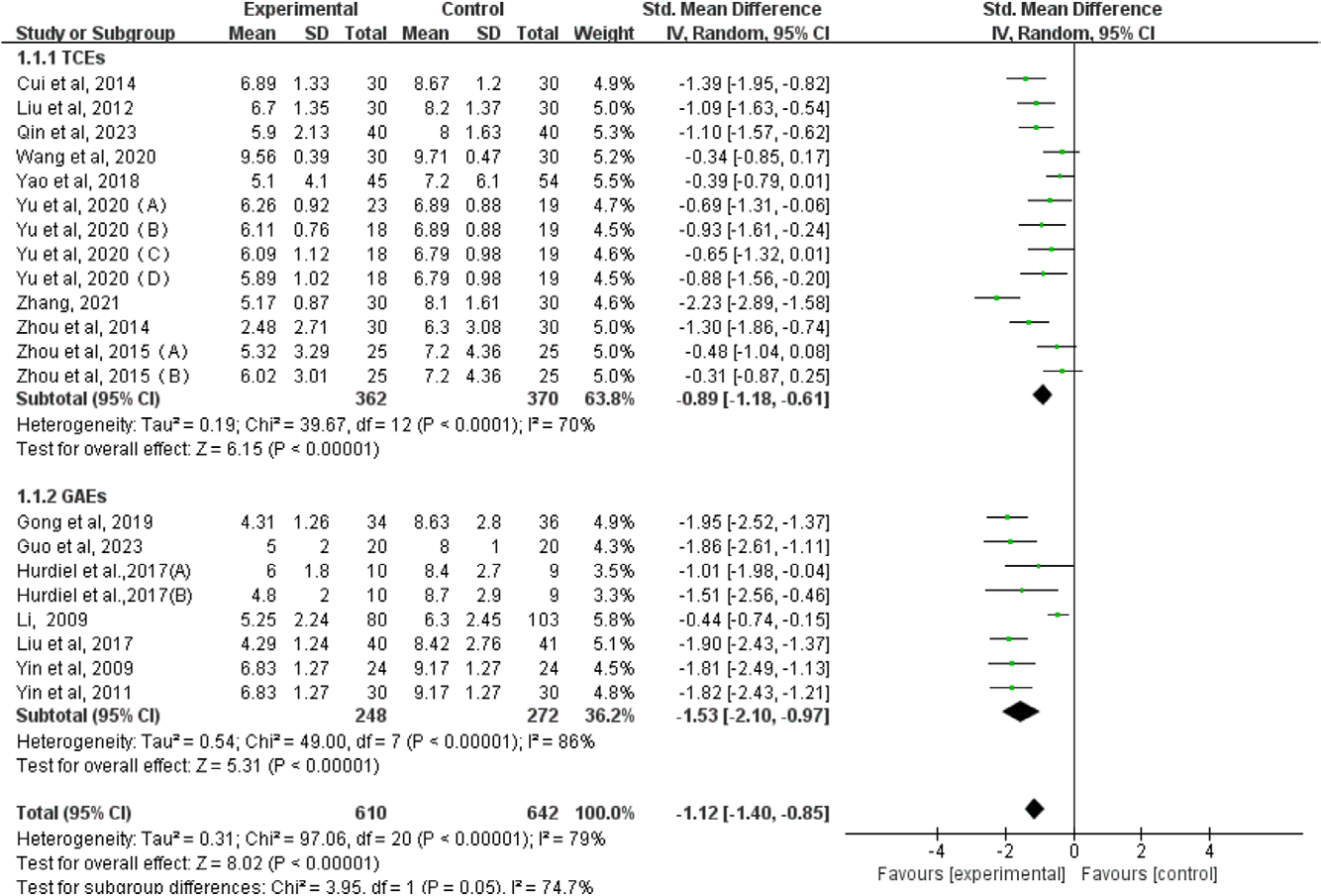
Forest plot for SMD of the Overall Effect between TCEs and GAEs for University Students. GAEs = general aerobic exercises, SMD = standard mean difference, TCEs = Traditional Chinese Exercises.

A total of 618 subjects were tested for global effects on data from the 13 included TCEs experiments (Fig. 6). The results showed an effect size of SMD = −0.89, (95% CI: −1.18 to −0.61; Z = 6. 15; *P* < .00001), and “-” represented the meaning of effective sleep improvement. Therefore, the results of Figure 6 show that TCEs can reduce the total PSQI score of university students, and achieve a large effect size, indicating that TCEs can effectively improve sleep. The overall heterogeneity test was performed for the included studies, revealing high heterogeneity among the studies (I^2^ = 70%, *P* < .00001). Consequently, a random-effects model was employed. A total of 520 subjects were tested to determine the global effects of the 8 GAEs included in the study (Fig. 6). The results revealed an effect size of SMD = −1.53 (95% CI: −2.10 ~ −0.97; Z = 5.31; *P* < .00001). These findings suggest that general GAEs can effectively reduce the total PSQI score of university students, indicating an improvement in sleep quality. The overall heterogeneity of the included studies was assessed using the I^2^ statistic, which indicated high heterogeneity among the studies (I^2^ = 86%, *P* < .00001). Consequently, a random-effects model was employed. A significant difference was found between the TCEs and GAEs groups (*P* = .05). The effect size of the GAEs group was found to be smaller than that of the TCEs group, indicating that GAEs had a greater impact on improving sleep in university students. It is important to note that due to the high degree of heterogeneity among the studies included in this meta-analysis, the overall effect size of this study may be influenced by various moderator variables.

#### 3.4.2. Seven dimensions of PSQI

The use of TCEs resulted in significant improvements in various aspects of sleep quality (Table 3). These included subjective sleep quality (SMD = −0.49, 95% CI: −0.53 to −0.40, *P* < .00001; 5 studies, n = 280), sleep duration (SMD = −0.55, 95% CI: −0.67 to −0.42, *P* < .00001; 5 studies, n = 280), sleep latency (SMD = −0.14, 95% CI: −0.21 to −0.08, *P* < .00001; 5 studies, n = 280), habitual sleep efficiency (SMD = −0.45, 95% CI: −0.51 to −0.38, *P* < .001; 5 studies, n = 220), sleep disturbance (SMD = −0.24, 95% CI: −0.31 to −0.16, *P* < .00001; 5 studies, n = 280), use of sleep medication (SMD = −0.14, 95% CI: −0.19 to −0.09, *P* < .00001; 4 studies, n = 280), and daytime dysfunction (SMD = −0.40, 95% CI: −0.46 to −0.34, *P* < .00001; 5 studies, n = 219). On the other hand, GAEs improved sleep quality across various measures (Table 3). Subjective sleep quality showed a significant improvement (SMD = −0.57, 95% CI: −0.68 to −0.45, *P* < .00001; 5 studies, n = 299). Sleep duration also increased (SMD = −0.64, 95% CI: −0.79 to −0.50, *P* < .00001; 5 studies, n = 299), while sleep latency decreased (SMD = −0.24, 95% CI: −0.38 to −0.10, *P* < .00001; 5 studies, n = 299). Habitual sleep efficiency improved as well (SMD = −0.33, 95% CI: −0.46 to −0.20, *P* < .001; 5 studies, n = 299). Furthermore, GAEs reduced sleep disturbance (SMD = −0.47, 95% CI: −0.58 to −0.36, *P* < .00001; 5 studies, n = 299), use of sleep medication (SMD = −0.18, 95% CI: −0.32 to −0.03, *P* < .00001; 4 studies, n = 299), and daytime dysfunction (SMD = −0.52, 95% CI: −0.66 to −0.39, *P* < .00001; 5 studies, n = 299).

**Table 3:** Seven dimensions of PSQI.

Subgroup analysis	Group	Heterogeneity test		Effect size and 95% confidence interval	Two-tailed test		Sample size	Number of documents
		I^2^ (%)	*P*		Z	*P*		
Quality of sleep	TCEs	39	.16	−0.46 [−0.53, −0.40]	14.53	<.00001	280	5
	GAEs	0	.58	−0.57 [−0.68, −0.45]	9.53	<.00001	299	5
Time to fall asleep	TCEs	70	.009	−0.55 [−0.67, −0.42]	8.68	<.00001	280	5
	GAEs	83	<.0001	−0.64 [−0.79, −0.50]	8.77	<.00001	299	5
Sleep duration	TCEs	45	.12	−0.14 [−0.21, −0.08]	4.39	<.0001	280	5
	GAEs	69	.02	−0.24 [−0.38, −0.10]	3.34	.0008	239	4
Sleep efficiency	TCEs	69	.002	−0.45 [−0.51, −0.38]	14.13	<.00001	220	4
	GAEs	60	.04	−0.33 [−0.46, −0.20]	5.04	<.00001	299	5
Sleep disturbance	TCEs	77	.002	−0.24 [−0.31, −0.16]	6.30	<.00001	280	5
	GAEs	96	<.00001	−0.47 [−0.58, −0.36]	8.63	<.00001	299	5
Hypnotic drugs	TCEs	68	.02	−0.14 [−0.19, −0.09]	5.32	<.00001	220	4
	GAEs	0	.68	−0.18 [−0.32, −0.03]	2.43	.01	219	4
Daytime disorders	TCEs	97	<.00001	−0.40 [−0.46, −0.34]	13.14	<.00001	280	5
	GAEs	44	.13	−0.52 [−0.66, −0.39]	7.46	<.00001	299	5

GAEs = general aerobic exercises, PSQI = Pittsburgh Sleep Quality Inventory, TCEs = Traditional Chinese Exercises.

#### 3.4.3. Subgroup analysis of GAEs on total PSQI score

The effect of exercise dose was further explored by subgroup analysis of 3 moderator variables: times per session, frequency per week, and period in the exercise protocol (Table 4). The subjects were divided into 2 groups based on the duration of each session: 40 to 60 minutes and 60 to 90 minutes. A subgroup analysis was performed on a total of 520 subjects from 8 RCTs articles that were included. The results indicated that the effect size observed in the 40 to 60 minutes group was greater than that in the 60 to 90 minutes group (SMD = −1.89; 95% CI: −2.19 to −1.59; Z = 12.31; *P* < .00001). The frequency per week was categorized into 2 groups: 2 sessions per week and 3 to 5 sessions per week. A subgroup analysis was conducted on the 8 included RCT articles, which revealed that the effect size was greater in the group that had 3 to 5 sessions per week compared to the group that had 2 sessions per week (SMD = −1.56; 95% CI: −2.33 to −0.80; Z = 4; *P* < .0001). The duration of the intervention was divided into 2 groups: 2 to 4 weeks and 6 to 18 weeks. A subgroup analysis was performed on 7 RCTs articles, including a total of 566 subjects. The findings indicated that the effect size of period was higher during the 2 to 4 weeks period compared to the 6 to 18 weeks period. (SMD = −1.85; 95% CI: −2.17 to −1.54; Z = 11.6; *P* < .00001).

**Table 4:** Results of subgroup analysis in GAEs program.

Subgroup analysis	Heterogeneity test			Groups	Effect size and 95% confidence interval	Two-tailed test		Study	Sample numbers
	I^2^ (%)		*P*			Z	*P*		
Times per session	86	0	.99	40~60 min	−1.89 [−2.19, −1.59]	12.31	<.00001	4	251
		81	.001	60~90 min	−1.15 [−1.93, −0.36]	2.86	=.004	4	269
Frequency per week	86	0	.40	2次	−1.53 [−2.05, −1.02]	5.80	<.00001	3	78
		91	<.00001	3~5次	−1.56 [−2.33, −0.80]	4.00	<.0001	5	442
Period	0	0	1.00	2~4周	−1.85 [−2.17, −1.54]	11.60	<.00001	4	229
		28	.25	6~18周	−1.61 [−2.17, −1.05]	5.63	<.0001	3	337

GAEs = general aerobic exercises.

#### 3.4.4. Subgroup analysis of TCEs on total PSQI score

The effect of exercise dose was further explored by subgroup analysis of 3 moderator variables: times per session, frequency per week, and period in the exercise protocol (Table 5). Times per session was divided into 2 groups: 30 to 60 minutes and 60 to 90 minutes. Subgroup analysis was conducted on a total of 612 subjects from the 11 included RCT articles. The results revealed that the effect size observed in the 60–90 minutes group was greater than that in the 60–90 minutes group (SMD = −1.37; 95% CI: −1.68 to −1.07; Z = 8.13; *P* < .00001). The frequency per week was categorized into 2 groups: 2 to 3 times per week and 5 times per week. Subgroup analysis was performed on the 11 included RCT articles involving a total of 622 subjects. The findings indicated that the effect size was higher for 2 to 3 times per week compared to 5 times per week (SMD = −2.34; 95% CI: −2.99 to −1.70; Z = 7.1; *P* < .00001). The duration was divided into 2 groups: 8 to 12 weeks and 16 to 24 weeks. Subgroup analysis was conducted on the 12 included RCT articles with a total of 672 subjects. The results showed that period produced an effect size greater at 8 to 12 weeks than at 16 to 24 weeks (SMD = −1.19; 95% CI: −1.47 to −0.92; Z = 8. 42; *P* < .00001).

**Table 5:** Results of subgroup analysis in TCEs program.

Subgroup analysis	Heterogeneity test			Groups	Effect size and 95% confidence interval	Two-tailed test		Study	Sample numbers
	I^2^ (%)		*P*			Z	*P*		
Times per session	68	46	.14	30~60min	−0.93 [−1.28, −0.57]	5.11	<.00001	4	238
		73	.001	60~90min	−1.37 [−1.68, −1.07]	8.13	<.00001	7	374
Frequency per week	86	45	<.00001	2~3次	−2.34 [−2.99, −1.70]	7.1	<.00001	4	289
		83	<.00001	5次	−0.52 [−0.68, −0.36]	6.19	<.00001	7	333
Period	85	73	.005	8~12周	−1.19 [−1.47, −0.92]	8.42	<.00001	5	279
		82	<.00001	16~24周	−0.37 [−0.56, −0.17]	3.72	=.0002	7	393

TCEs = Traditional Chinese Exercises.

## 4. Discussion

### 4.1. Effects of TCEs and GAEs on sleep disorders in university students

The study results reveal that both TCEs and GAEs positively impact the sleep quality of university students (SMD = −1.12, *P* < .00001). Moreover, aerobic exercise appears to be more effective than TCEs.

These findings align with previous research. Liu et al suggested that TCEs could improve sleep quality in specific populations, holding clinical significance for certain groups.^[38]^ Similarly, Wu et al showed that both TCEs and GAEs, as non-pharmacological and complementary interventions, can enhance sleep quality in older adults, potentially offering clinical benefits.^[39]^ In contrast to the results of this study, Marian et al conducted a control experiment that demonstrated yoga exercise did not result in an enhancement of sleep quality in university students.^[40]^ The authors explained this difference by pointing out the high intensity of exercise in the experimental group, whereas the control group continued their regular daily physical activity. The studies included in our research focused on moderate-intensity aerobic exercise and TCEs. The intervention measures in the control group were carefully regulated and designed to be more accessible for ordinary university students without training experience. As a result, the findings obtained are considered to be more reliable.

The study demonstrates that exercise has a significant impact on sleep quality, with effects primarily attributed to physiological and psychological mechanisms. Physiologically, exercise enhances nervous system function by increasing neurotransmitters, regulating body temperature, promoting melatonin secretion, and facilitating quick entry into sleep.^[41]^ These factors play a crucial role in regulating biological rhythms, reducing fatigue, and promoting restful sleep.^[16,42,43]^ Psychologically, exercise helps alleviate stress, improve sleep quality by transforming negative emotions, and enhance mental well-being.^[44]^ Additionally, regular physical activity can aid university students in correcting unhealthy lifestyles and habits.

### 4.2. The comparative analysis of the effects of TCEs and GAEs on improving sleep quality in university students

Our study found that GAEs (SMD = −1.53, *P* < .00001) were more effective than TCEs (SMD = −0.89, *P* < .00001) in improving sleep quality among university students. This contrasts with the findings of previous research, which did not provide sufficient evidence to demonstrate the difference in the impact on sleep quality between TCEs practices and aerobic exercise.^[39]^ Our study participants were all college students without any other health conditions.

Previous research has predominantly focused on older individuals with conditions such as stroke, depression, and cancer, who often experience fragmented sleep and may have additional health issues like sleep-disordered breathing, exacerbating sleep problems. This suggests that different types of exercise could have varying effects on sleep quality depending on the population. Moreover, prior studies included participants with diverse medical conditions, potentially influencing results when merging data from different patients.^[19,20]^ Additionally, the quality of the RCTs included in the study could also influence its findings.

The mechanisms by which TCEs and aerobic exercise improve sleep may differ. TCEs such as the Five Animal Play, Eight Duan Jin, Qi Gong, and Yi Jin Jing emphasize various body movements combined with breathing techniques and psychological regulation to fully exploit the body potential for improving sleep.^[45,46]^ These exercises focus on promoting tranquility, inner peace, and joy, leading to a relaxed and comfortable state of mind conducive to better sleep quality. The incorporation of soothing music enhances relaxation during exercise, while increased plasma melatonin concentration further enhances sleep quality. During GAEs, the pituitary gland releases endorphins, which are morphine-like hormones. Endorphin levels in the body can remain elevated during prolonged aerobic exercise sessions lasting more than 60 minutes.^[18]^ Psychologists argue that endorphins produced during aerobic exercise serve as potent physiological sedatives.^[47]^ Consequently, the reduction or disappearance of insomnia symptoms in individuals who engage in regular physical activity is attributed to the effects of endorphins.^[48]^ Moreover, GAEs induces a sense of fatigue in practitioners after training. Reduced brain excitability inhibits substance secretion, accelerating sleep onset time, promoting deep sleep, and quickly alleviating fatigue, thereby establishing a positive cycle of improved sleep.^[49]^ Research indicates that aerobic calisthenics can enhance the neuro-endocrine function of the body, weaken the function of the serotonergic system in the central nervous system, and stimulate an increase in brain-derived neurotrophic factor through activation of the dopaminergic system.^[50]^ This plays a crucial role in regulating brain function and mood disorders, leading to stress alleviation, reduced anxiety and depression levels, minimized dream interruptions, and alleviated sleep disorders.^[51]^ Additionally, aerobic exercise can regulate the biological rhythm of university students, thereby comprehensively enhancing sleep quality. Human movement is directly or indirectly regulated by the nervous system, which plays a central role in controlling bodily movements.^[17]^ Moderate-intensity GAEs increases the speed of blood circulation throughout the body, enhances blood flow to the brain, and facilitates the recovery of cerebral cortex function.^[52]^ Consequently, the cerebral cortex emits bioelectrical signals that act on skeletal muscles and innervate their activity.^[53]^ When skeletal muscles are fatigued or exhausted, impulses to the brain decrease, inducing a state of calmness and gradually facilitating the transition into sleep.

### 4.3. Subgroup analysis of the effect of GAEs on sleep disorders in university students

In this study, subgroup analysis of conditioning variables for exercise regimens (times per session, frequency per week, period) revealed that each exercise produced the largest effect size at 40 to 60 minutes, and exercise 3 to 5 times a week improved sleep quality most significantly in university students, and continuous exercise for more than 2 to 4 weeks could achieve the best effect in improving sleep. Kredlow et al conducted a meta-analysis investigating the effects of acute and regular exercise on sleep, incorporating a range of outcomes and moderator variables.^[54]^ The results revealed that engaging in regular exercise 3~5 times per week had small beneficial effects on total sleep time and sleep efficiency, small to moderate beneficial effects on sleep onset latency, and moderate beneficial effects on sleep quality. These findings also support the results of our subgroup analysis.^[54]^ Kubitz et al also indicates that long-term exercise is more beneficial for improving sleep quality than acute exercise. This is consistent with our finding that GAEs for more than 4 weeks enhances sleep quality.^[55]^ It is worth noting that there was no significant difference between the groups with continuous exercise for 2 to 4 weeks and 5 to 18 weeks (*P* > .05), and there was only one literature with intervention for 2 weeks, which easily produced wrong conclusions, while most studies focused on more than 4 weeks and were large effect sizes, SMD = −1.85, which was significant (*P* < .00001), which was also consistent with the number of exercises recommended by the World Health Organization.^[55]^ Thus, the effect of sustained exercise for more than 4 weeks was significant. Because the physiological effects of moderate intensity GAEs and physical and mental exercise on the body are mild, regular GAEs is effective in improving the sleep quality of university students.

### 4.4. Limitations and prospects

This study is a meta-analysis of RCTs. However, it should be noted that certain cohort trials with large sample sizes and extensive data were not included. The overall effect size of this study and the results of subgroup analysis of moderator variables depend on the quality and quantity of the included articles. Because there are inevitable factors in the retrieval process, such as: foreign language literatures with abstract without full text, English literatures that cannot be obtained and ask for fruitless results for many times, etc. Ultimately, only 2 data from one English article were included in this study. In addition, future assessment of sleep quality can be measured using a data-based instrument, and data obtained by brain waves, sleep machines are more credible than subjective evaluation scales. In terms of subgroup analysis of regulatory variables, further research can be conducted from university students with different baseline sleep status, different genders, personality characteristics, and different grades in the future to make exercise prescription more accurate and effective for improving university students’ sleep quality. This study only explores the single item GAEs, the form of group club GAEs on sleep improvement may be more significant research, the future club group exercise on sleep status can be studied.

## 5. Conclusion and suggesting

GAEs and TCEs can improve the sleep quality of university students and GAEs improve the sleep of university students better than TCEs. Regular GAEs program using 40 to 60 minutes/time, 3 to 5 times/week for 4 weeks was the best to improve sleep in university students. We suggest that aerobic training 3 to 5 times a week, 40 to 60 minutes each time, lasting for more than 4 weeks is an effective method to improve the sleep quality of university students with sleep disorders.

## Acknowledgements

We thank each of the authors for their contributions

## Author contributions

**Conceptualization:** Haiting Zhai.

**Data curation:** Zhihui Yang.

**Formal analysis:** Zhihui Yang.

**Project administration:** Zhihui Yang.

**Software:** Zhihui Yang, Zhiwei Yang, Boxuan Ning.

**Supervision:** Zhihui Yang.

**Validation:** Zhihui Yang.

**Writing – original draft:** Zhihui Yang.

**Writing – review & editing:** Zhihui Yang, Haiting Zhai.
